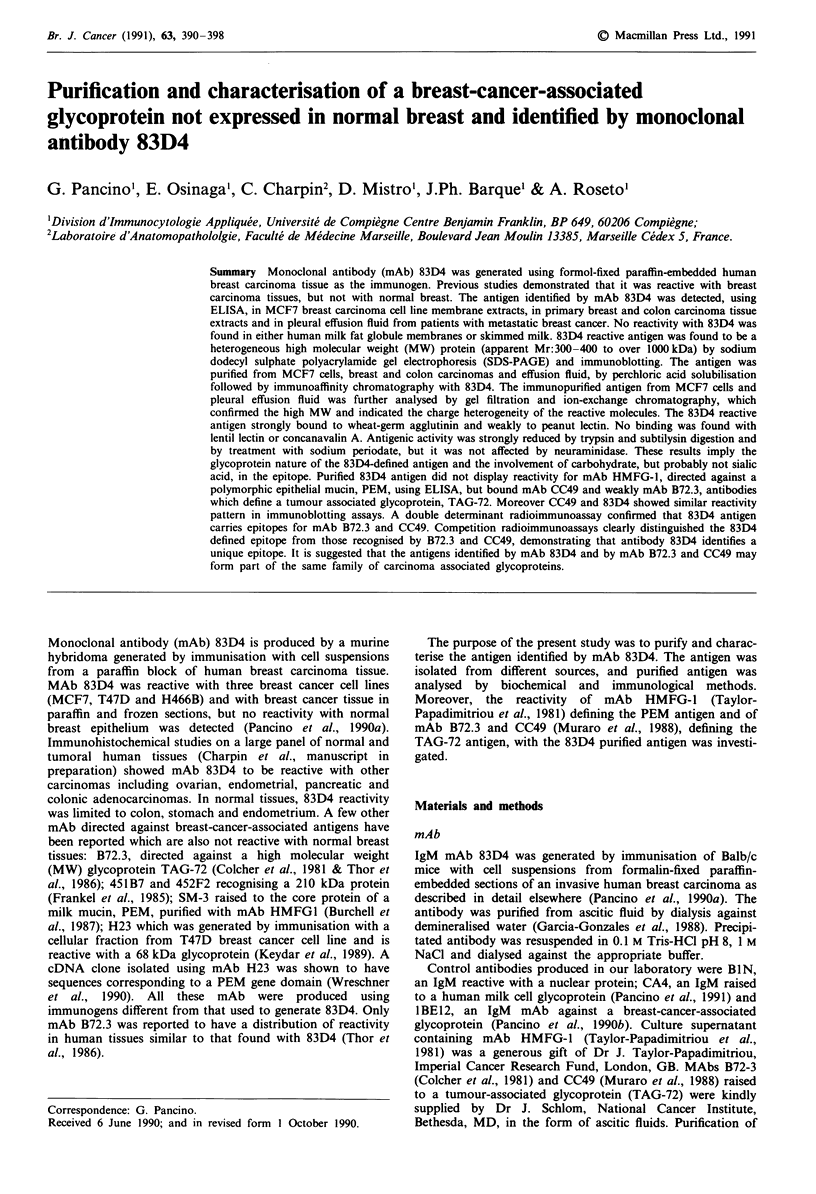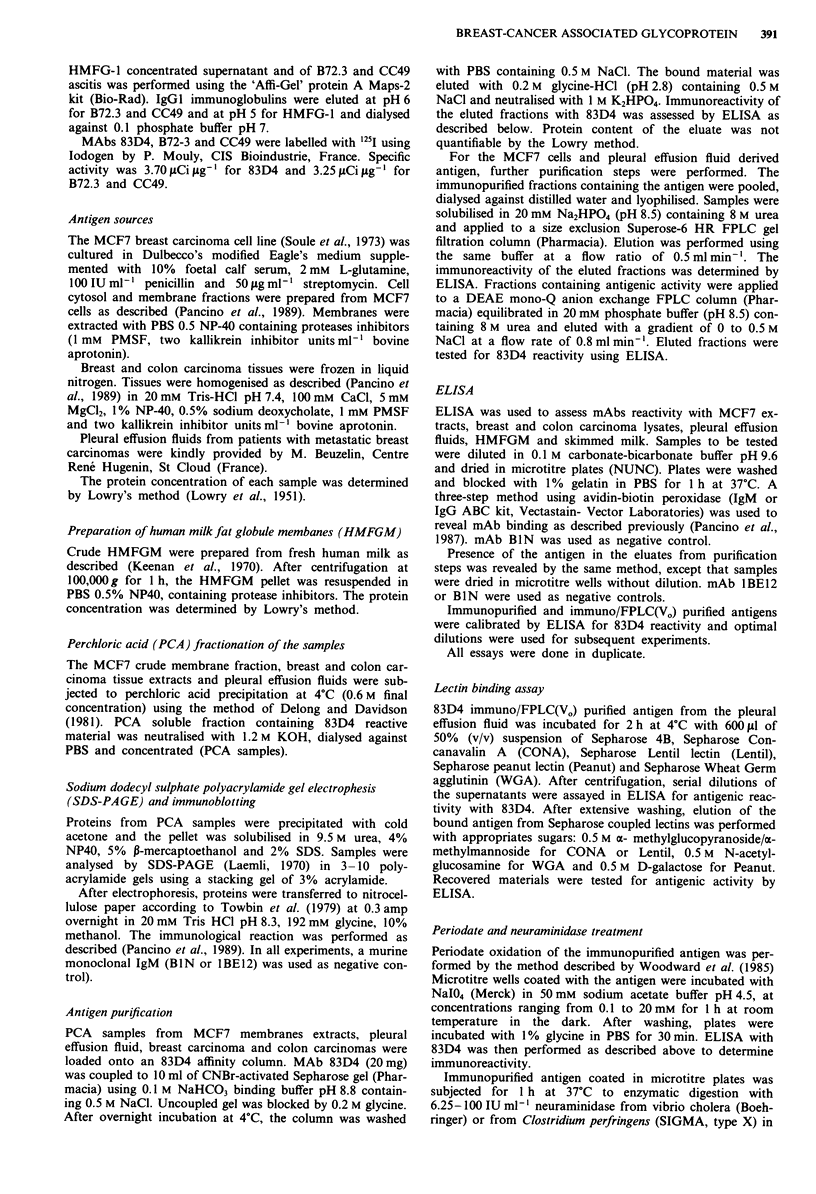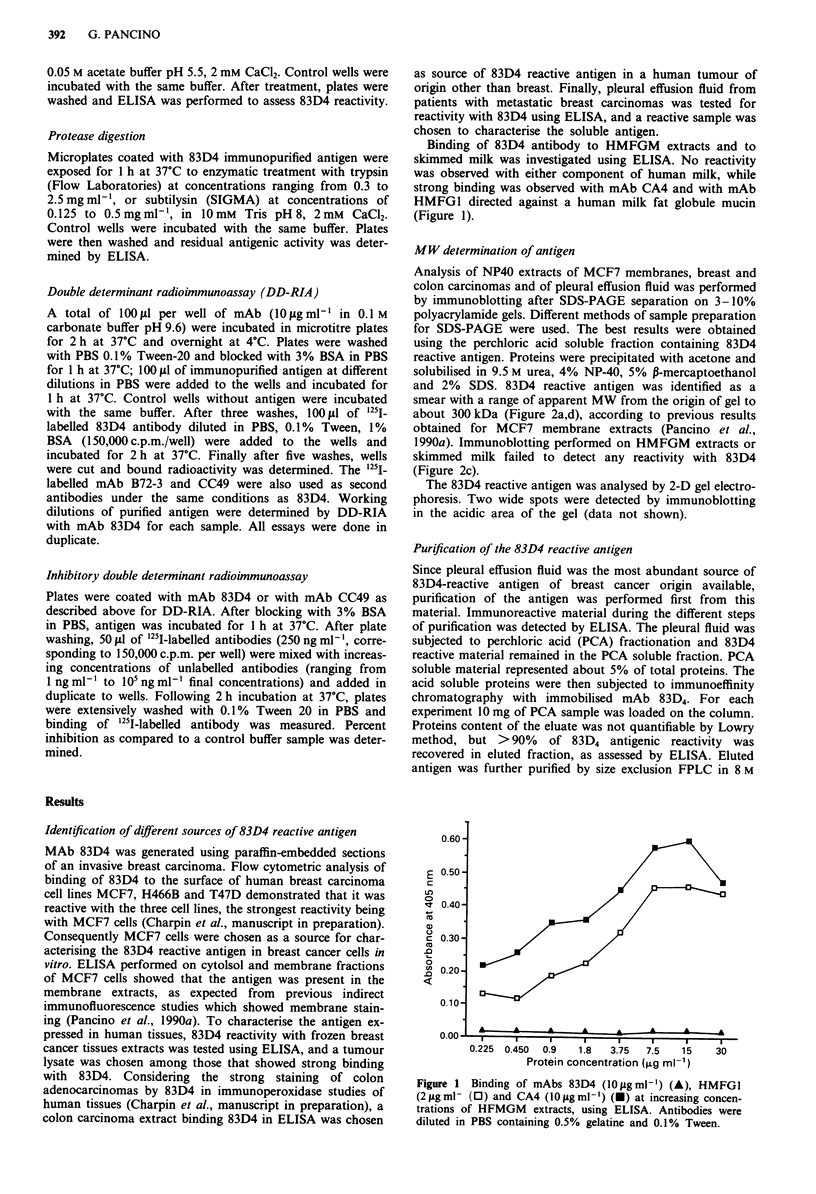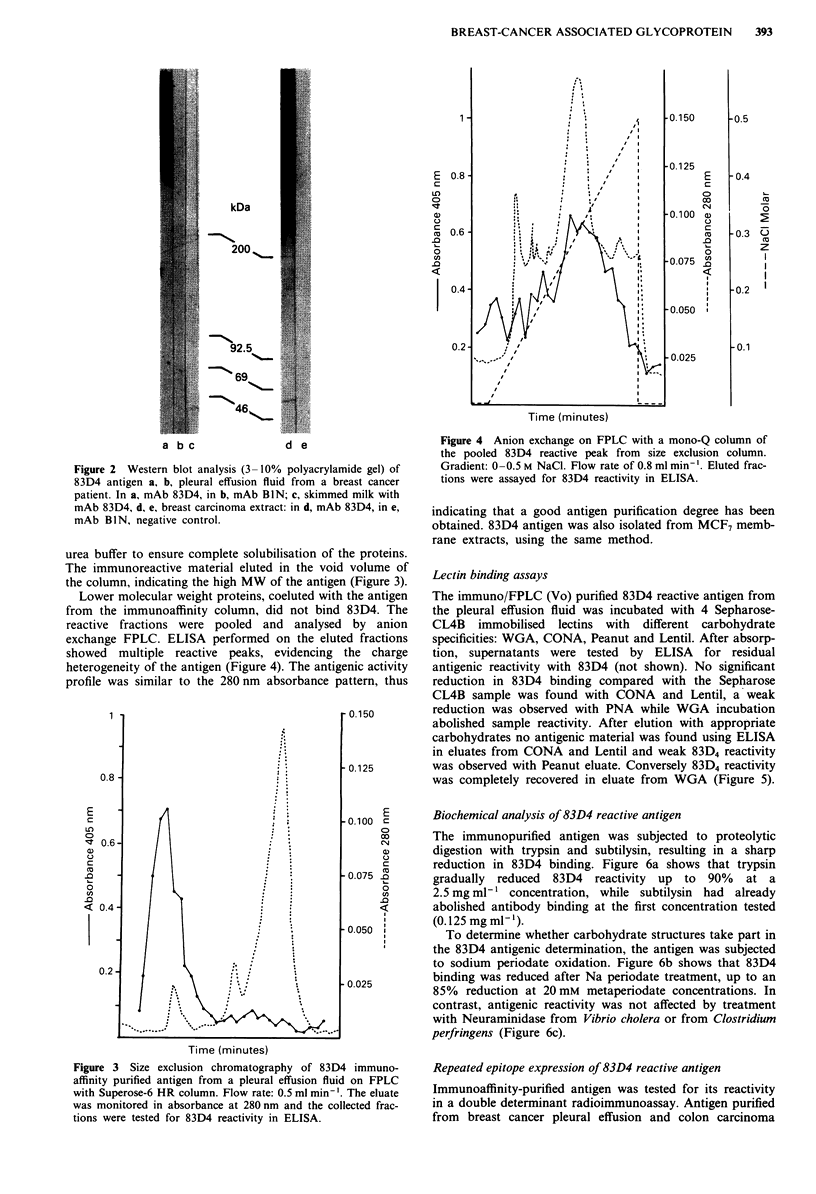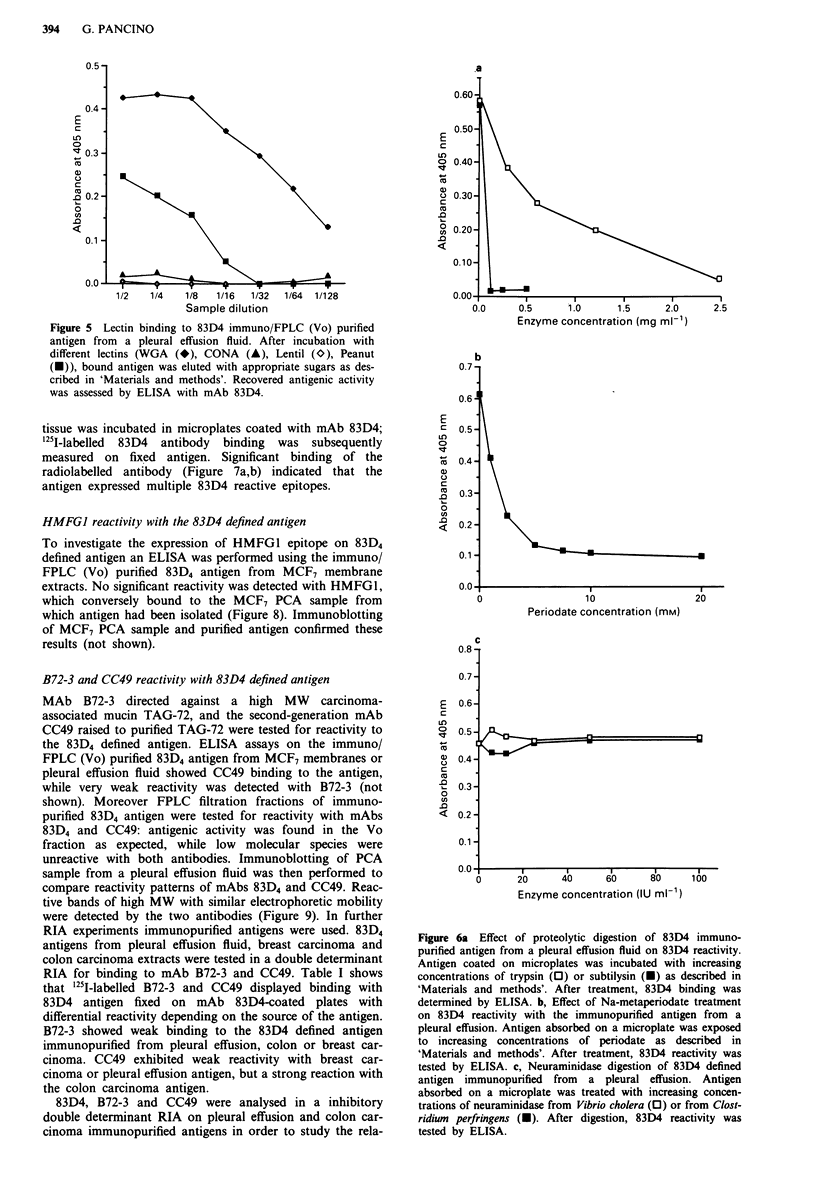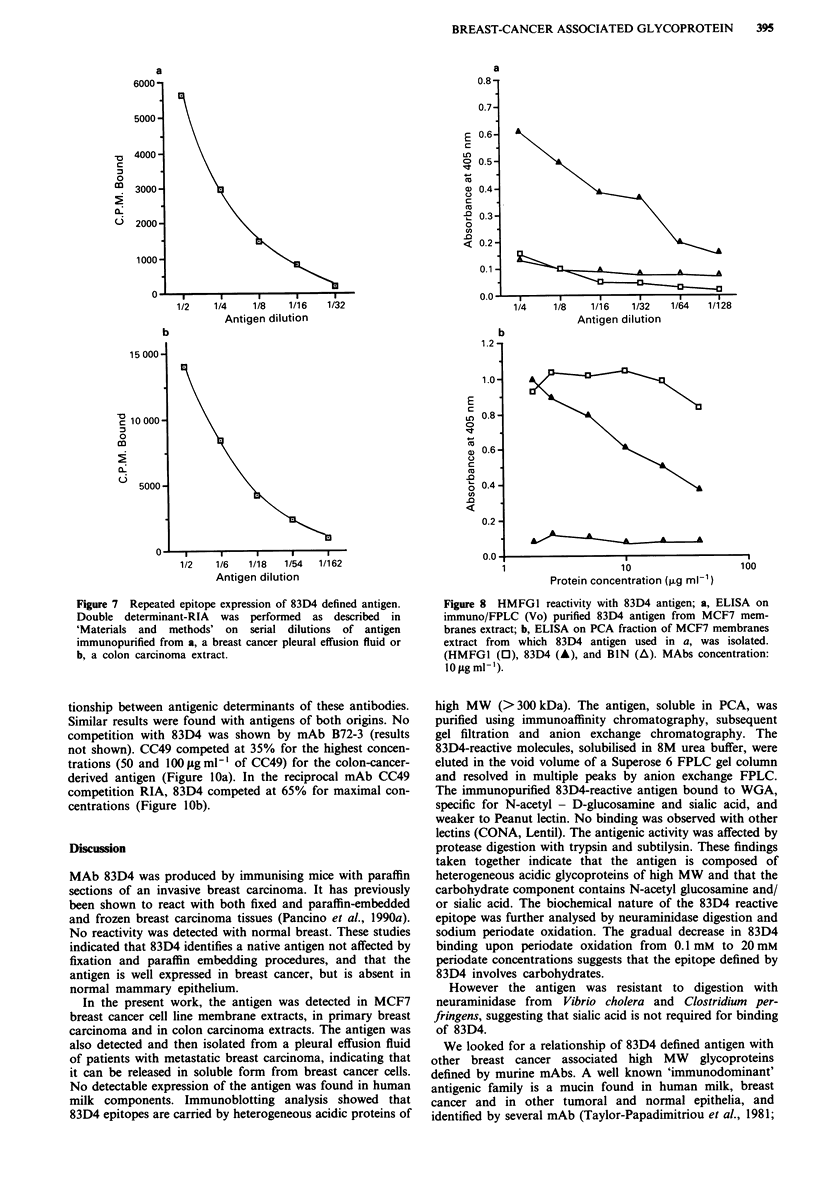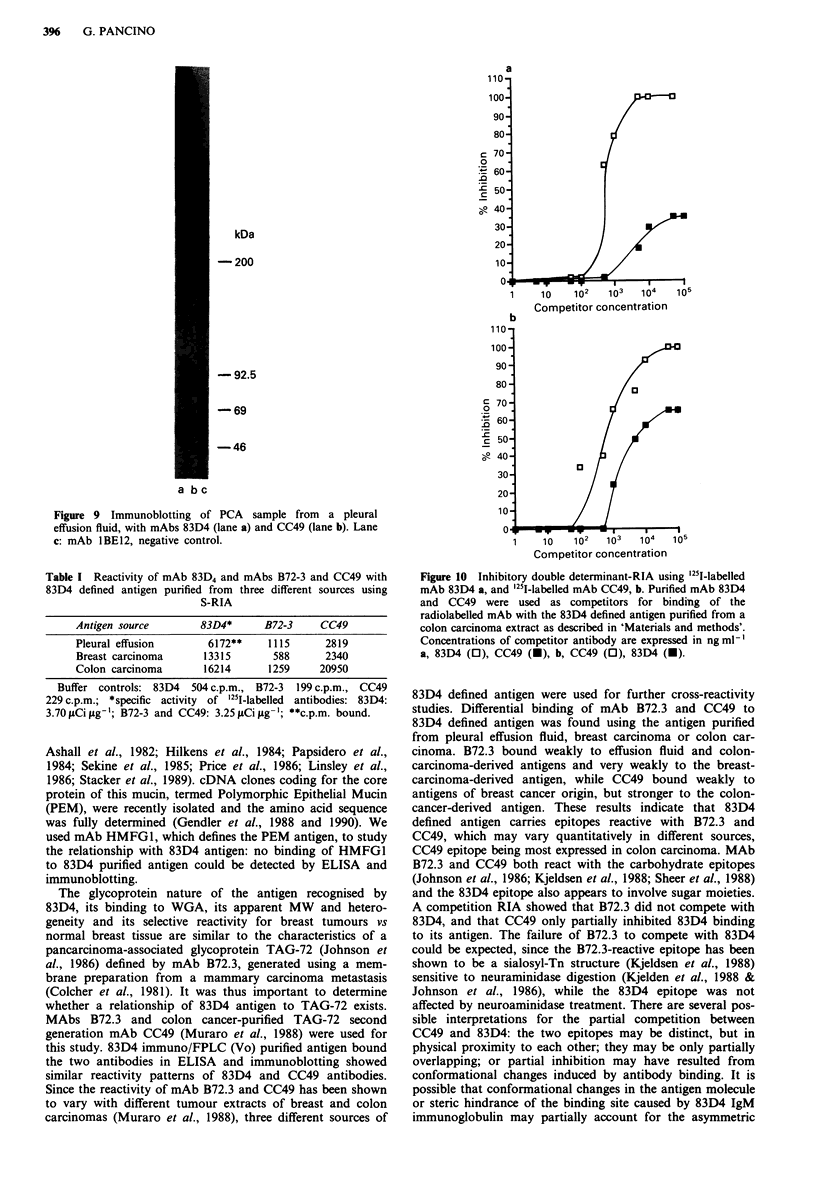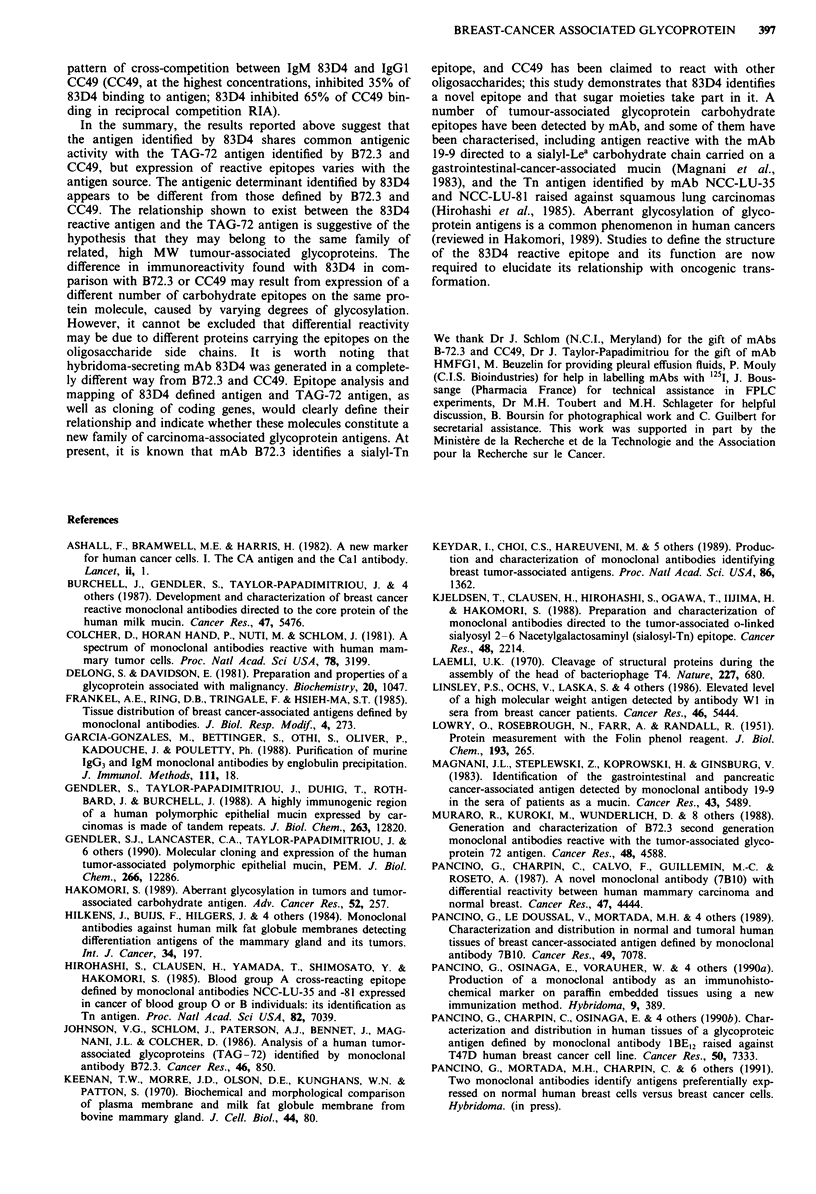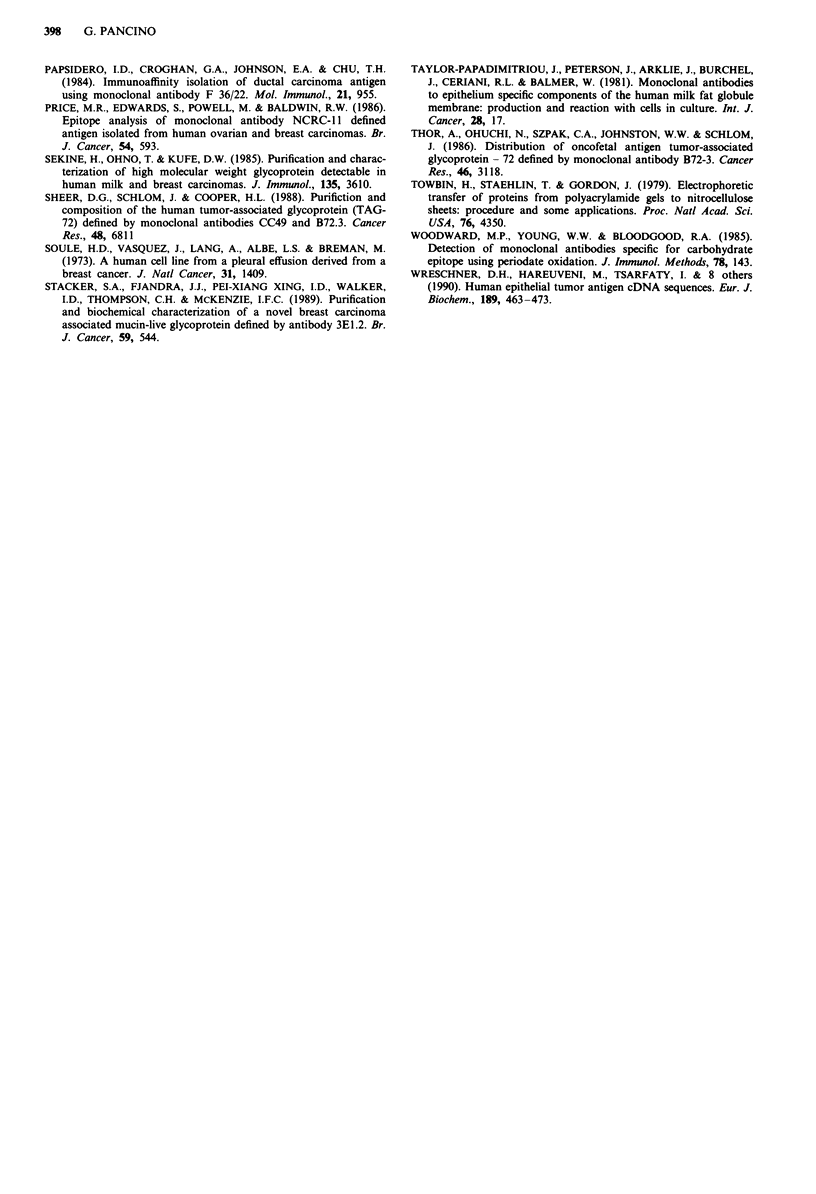# Purification and characterisation of a breast-cancer-associated glycoprotein not expressed in normal breast and identified by monoclonal antibody 83D4.

**DOI:** 10.1038/bjc.1991.91

**Published:** 1991-03

**Authors:** G. Pancino, E. Osinaga, C. Charpin, D. Mistro, J. P. Barque, A. Roseto

**Affiliations:** Division d'Immunocytologie Appliquée, Université de Compiègne Centre Benjamin Franklin, France.

## Abstract

**Images:**


Br. J. Cancer (1991), 63, 390-398 © Macmillan Press Ltd., 1991

### Purification and characterisation of a breast-cancer-associated glycoprotein not expressed in normal breast and identified by monoclonal antibody 83D4

G. Pancino[1], E. Osinaga[1], C. Charpin[2], D. Mistro[1], J.Ph. Barque[1] & A. Roseto[1]

[1]*Division d'Immunocytologie Appliquée, Université de Compiègne Centre Benjamin Franklin, BP 649, 60206 Compiègne;* [2]*Laboratoire d'Anatomopatholoigie, Faculté de Médecine Marseille, Boulevard Jean Moulin 13385, Marseille Cédex 5, France.*

**Summary** Monoclonal antibody (mAb) 83D4 was generated using formol-fixed paraffin-embedded human breast carcinoma tissue as the immunogen. Previous studies demonstrated that it was reactive with breast carcinoma tissues, but not with normal breast. The antigen identified by mAb 83D4 was detected, using ELISA, in MCF7 breast carcinoma cell line membrane extracts, in primary breast and colon carcinoma tissue extracts and in pleural effusion fluid from patients with metastatic breast cancer. No reactivity with 83D4 was found in either human milk fat globule membranes or skimmed milk. 83D4 reactive antigen was found to be a heterogeneous high molecular weight (MW) protein (apparent Mr:300-400 to over 1000 kDa) by sodium dodecyl sulphate polyacrylamide gel electrophoresis (SDS-PAGE) and immunoblotting. The antigen was purified from MCF7 cells, breast and colon carcinomas and effusion fluid, by perchloric acid solubilisation followed by immunoaffinity chromatography with 83D4. The immunopurified antigen from MCF7 cells and pleural effusion fluid was further analysed by gel filtration and ion-exchange chromatography, which confirmed the high MW and indicated the charge heterogeneity of the reactive molecules. The 83D4 reactive antigen strongly bound to wheat-germ agglutinin and weakly to peanut lectin. No binding was found with lentil lectin or concanavalin A. Antigenic activity was strongly reduced by trypsin and subtilysin digestion and by treatment with sodium periodate, but it was not affected by neuraminidase. These results imply the glycoprotein nature of the 83D4-defined antigen and the involvement of carbohydrate, but probably not sialic acid, in the epitope. Purified 83D4 antigen did not display reactivity for mAb HMFG-1, directed against a polymorphic epithelial mucin, PEM, using ELISA, but bound mAb CC49 and weakly mAb B72.3, antibodies which define a tumour associated glycoprotein, TAG-72. Moreover CC49 and 83D4 showed similar reactivity pattern in immunoblotting assays. A double determinant radioimmunoassay confirmed that 83D4 antigen carries epitopes for mAb B72.3 and CC49. Competition radioimmunoassays clearly distinguished the 83D4 defined epitope from those recognised by B72.3 and CC49, demonstrating that antibody 83D4 identifies a unique epitope. It is suggested that the antigens identified by mAb 83D4 and by mAb B72.3 and CC49 may form part of the same family of carcinoma associated glycoproteins.

Monoclonal antibody (mAb) 83D4 is produced by a murine hybridoma generated by immunisation with cell suspensions from a paraffin block of human breast carcinoma tissue. MAb 83D4 was reactive with three breast cancer cell lines (MCF7, T47D and H466B) and with breast cancer tissue in paraffin and frozen sections, but no reactivity with normal breast epithelium was detected (Pancino *et al.*, 1990*a*). Immunohistochemical studies on a large panel of normal and tumoral human tissues (Charpin *et al.*, manuscript in preparation) showed mAb 83D4 to be reactive with other carcinomas including ovarian, endometrial, pancreatic and colonic adenocarcinomas. In normal tissues, 83D4 reactivity was limited to colon, stomach and endometrium. A few other mAb directed against breast-cancer-associated antigens have been reported which are also not reactive with normal breast tissues: B72.3, directed against a high molecular weight (MW) glycoprotein TAG-72 (Colcher *et al.*, 1981 & Thor *et al.*, 1986); 451B7 and 452F2 recognising a 210 kDa protein (Frankel *et al.*, 1985); SM-3 raised to the core protein of a milk mucin, PEM, purified with mAb HMFG1 (Burchell *et al.*, 1987); H23 which was generated by immunisation with a cellular fraction from T47D breast cancer cell line and is reactive with a 68 kDa glycoprotein (Keydar *et al.*, 1989). A cDNA clone isolated using mAb H23 was shown to have sequences corresponding to a PEM gene domain (Wreschner *et al.*, 1990). All these mAb were produced using immunogens different from that used to generate 83D4. Only mAb B72.3 was reported to have a distribution of reactivity in human tissues similar to that found with 83D4 (Thor *et al.*, 1986).

Correspondence: G. Pancino.
Received 6 June 1990; and in revised form 1 October 1990.

The purpose of the present study was to purify and characterise the antigen identified by mAb 83D4. The antigen was isolated from different sources, and purified antigen was analysed by biochemical and immunological methods. Moreover, the reactivity of mAb HMFG-1 (Taylor-Papadimitriou *et al.*, 1981) defining the PEM antigen and of mAb B72.3 and CC49 (Muraro *et al.*, 1988), defining the TAG-72 antigen, with the 83D4 purified antigen was investigated.

### Materials and methods

### *mAb*

IgM mAb 83D4 was generated by immunisation of Balb/c mice with cell suspensions from formalin-fixed paraffin-embedded sections of an invasive human breast carcinoma as described in detail elsewhere (Pancino *et al.*, 1990*a*). The antibody was purified from ascitic fluid by dialysis against demineralised water (Garcia-Gonzales *et al.*, 1988). Precipitated antibody was resuspended in 0.1 M Tris-HCl pH 8, 1 M NaCl and dialysed against the appropriate buffer.

Control antibodies produced in our laboratory were B1N, an IgM reactive with a nuclear protein; CA4, an IgM raised to a human milk cell glycoprotein (Pancino *et al.*, 1991) and 1BE12, an IgM mAb against a breast-cancer-associated glycoprotein (Pancino *et al.*, 1990*b*). Culture supernatant containing mAb HMFG-1 (Taylor-Papadimitriou *et al.*, 1981) was a generous gift of Dr J. Taylor-Papadimitriou, Imperial Cancer Research Fund, London, GB. MAbs B72-3 (Colcher *et al.*, 1981) and CC49 (Muraro *et al.*, 1988) raised to a tumour-associated glycoprotein (TAG-72) were kindly supplied by Dr J. Schlom, National Cancer Institute, Bethesda, MD, in the form of ascitic fluids. Purification of

HMFG-1 concentrated supernatant and of B72.3 and CC49 ascitis was performed using the 'Affi-Gel' protein A Maps-2 kit (Bio-Rad). IgG1 immunoglobulins were eluted at pH 6 for B72.3 and CC49 and at pH 5 for HMFG-1 and dialysed against 0.1 phosphate buffer pH 7.

MAbs 83D4, B72-3 and CC49 were labelled with $^{125}I$ using Iodogen by P. Mouly, CIS Bioindustrie, France. Specific activity was 3.70 $\mu$Ci $\mu g^{-1}$ for 83D4 and 3.25 $\mu$Ci $\mu g^{-1}$ for B72.3 and CC49.

*Antigen sources*

The MCF7 breast carcinoma cell line (Soule *et al.*, 1973) was cultured in Dulbecco's modified Eagle's medium supplemented with 10% foetal calf serum, 2 mM L-glutamine, 100 IU $ml^{-1}$ penicillin and 50 $\mu g\ ml^{-1}$ streptomycin. Cell cytosol and membrane fractions were prepared from MCF7 cells as described (Pancino *et al.*, 1989). Membranes were extracted with PBS 0.5 NP-40 containing proteases inhibitors (1 mM PMSF, two kallikrein inhibitor units $ml^{-1}$ bovine aprotonin).

Breast and colon carcinoma tissues were frozen in liquid nitrogen. Tissues were homogenised as described (Pancino *et al.*, 1989) in 20 mM Tris-HCl pH 7.4, 100 mM CaCl, 5 mM $MgCl_2$, 1% NP-40, 0.5% sodium deoxycholate, 1 mM PMSF and two kallikrein inhibitor units $ml^{-1}$ bovine aprotonin.

Pleural effusion fluids from patients with metastatic breast carcinomas were kindly provided by M. Beuzelin, Centre René Hugenin, St Cloud (France).

The protein concentration of each sample was determined by Lowry's method (Lowry *et al.*, 1951).

*Preparation of human milk fat globule membanes (HMFGM)*

Crude HMFGM were prepared from fresh human milk as described (Keenan *et al.*, 1970). After centrifugation at 100,000 *g* for 1 h, the HMFGM pellet was resuspended in PBS 0.5% NP40, containing protease inhibitors. The protein concentration was determined by Lowry's method.

*Perchloric acid (PCA) fractionation of the samples*

The MCF7 crude membrane fraction, breast and colon carcinoma tissue extracts and pleural effusion fluids were subjected to perchloric acid precipitation at 4°C (0.6 M final concentration) using the method of Delong and Davidson (1981). PCA soluble fraction containing 83D4 reactive material was neutralised with 1.2 M KOH, dialysed against PBS and concentrated (PCA samples).

*Sodium dodecyl sulphate polyacrylamide gel electrophesis (SDS-PAGE) and immunoblotting*

Proteins from PCA samples were precipitated with cold acetone and the pellet was solubilised in 9.5 M urea, 4% NP40, 5% $\beta$-mercaptoethanol and 2% SDS. Samples were analysed by SDS-PAGE (Laemli, 1970) in 3–10 polyacrylamide gels using a stacking gel of 3% acrylamide.

After electrophoresis, proteins were transferred to nitrocellulose paper according to Towbin *et al.* (1979) at 0.3 amp overnight in 20 mM Tris HCl pH 8.3, 192 mM glycine, 10% methanol. The immunological reaction was performed as described (Pancino *et al.*, 1989). In all experiments, a murine monoclonal IgM (B1N or 1BE12) was used as negative control).

*Antigen purification*

PCA samples from MCF7 membranes extracts, pleural effusion fluid, breast carcinoma and colon carcinomas were loaded onto an 83D4 affinity column. MAb 83D4 (20 mg) was coupled to 10 ml of CNBr-activated Sepharose gel (Pharmacia) using 0.1 M $NaHCO_3$ binding buffer pH 8.8 containing 0.5 M NaCl. Uncoupled gel was blocked by 0.2 M glycine. After overnight incubation at 4°C, the column was washed with PBS containing 0.5 M NaCl. The bound material was eluted with 0.2 M glycine-HCl (pH 2.8) containing 0.5 M NaCl and neutralised with 1 M $K_2HPO_4$. Immunoreactivity of the eluted fractions with 83D4 was assessed by ELISA as described below. Protein content of the eluate was not quantifiable by the Lowry method.

For the MCF7 cells and pleural effusion fluid derived antigen, further purification steps were performed. The immunopurified fractions containing the antigen were pooled, dialysed against distilled water and lyophilised. Samples were solubilised in 20 mM $Na_2HPO_4$ (pH 8.5) containing 8 M urea and applied to a size exclusion Superose-6 HR FPLC gel filtration column (Pharmacia). Elution was performed using the same buffer at a flow ratio of 0.5 ml $min^{-1}$. The immunoreactivity of the eluted fractions was determined by ELISA. Fractions containing antigenic activity were applied to a DEAE mono-Q anion exchange FPLC column (Pharmacia) equilibrated in 20 mM phosphate buffer (pH 8.5) containing 8 M urea and eluted with a gradient of 0 to 0.5 M NaCl at a flow rate of 0.8 ml $min^{-1}$. Eluted fractions were tested for 83D4 reactivity using ELISA.

*ELISA*

ELISA was used to assess mAbs reactivity with MCF7 extracts, breast and colon carcinoma lysates, pleural effusion fluids, HMFGM and skimmed milk. Samples to be tested were diluted in 0.1 M carbonate-bicarbonate buffer pH 9.6 and dried in microtitre plates (NUNC). Plates were washed and blocked with 1% gelatin in PBS for 1 h at 37°C. A three-step method using avidin-biotin peroxidase (IgM or IgG ABC kit, Vectastain- Vector Laboratories) was used to reveal mAb binding as described previously (Pancino *et al.*, 1987). mAb B1N was used as negative control.

Presence of the antigen in the eluates from purification steps was revealed by the same method, except that samples were dried in microtitre wells without dilution. mAb 1BE12 or B1N were used as negative controls.

Immunopurified and immuno/FPLC($V_o$) purified antigens were calibrated by ELISA for 83D4 reactivity and optimal dilutions were used for subsequent experiments.

All essays were done in duplicate.

*Lectin binding assay*

83D4 immuno/FPLC($V_o$) purified antigen from the pleural effusion fluid was incubated for 2 h at 4°C with 600 $\mu$l of 50% (v/v) suspension of Sepharose 4B, Sepharose Concanavalin A (CONA), Sepharose Lentil lectin (Lentil), Sepharose peanut lectin (Peanut) and Sepharose Wheat Germ agglutinin (WGA). After centrifugation, serial dilutions of the supernatants were assayed in ELISA for antigenic reactivity with 83D4. After extensive washing, elution of the bound antigen from Sepharose coupled lectins was performed with appropriates sugars: 0.5 M $\alpha$- methylglucopyranoside/$\alpha$-methylmannoside for CONA or Lentil, 0.5 M N-acetyl-glucosamine for WGA and 0.5 M D-galactose for Peanut. Recovered materials were tested for antigenic activity by ELISA.

*Periodate and neuraminidase treatment*

Periodate oxidation of the immunopurified antigen was performed by the method described by Woodward *et al.* (1985) Microtitre wells coated with the antigen were incubated with $NaI0_4$ (Merck) in 50 mM sodium acetate buffer pH 4.5, at concentrations ranging from 0.1 to 20 mM for 1 h at room temperature in the dark. After washing, plates were incubated with 1% glycine in PBS for 30 min. ELISA with 83D4 was then performed as described above to determine immunoreactivity.

Immunopurified antigen coated in microtitre plates was subjected for 1 h at 37°C to enzymatic digestion with 6.25–100 IU $ml^{-1}$ neuraminidase from vibrio cholera (Boehringer) or from *Clostridium perfringens* (SIGMA, type X) in

0.05 M acetate buffer pH 5.5, 2 mM $CaCl_2$. Control wells were incubated with the same buffer. After treatment, plates were washed and ELISA was performed to assess 83D4 reactivity.

*Protease digestion*

Microplates coated with 83D4 immunopurified antigen were exposed for 1 h at 37°C to enzymatic treatment with trypsin (Flow Laboratories) at concentrations ranging from 0.3 to 2.5 mg $ml^{-1}$, or subtilysin (SIGMA) at concentrations of 0.125 to 0.5 mg $ml^{-1}$, in 10 mM Tris pH 8, 2 mM $CaCl_2$. Control wells were incubated with the same buffer. Plates were then washed and residual antigenic activity was determined by ELISA.

*Double determinant radioimmunoassay (DD-RIA)*

A total of 100 µl per well of mAb (10 µg $ml^{-1}$ in 0.1 M carbonate buffer pH 9.6) were incubated in microtitre plates for 2 h at 37°C and overnight at 4°C. Plates were washed with PBS 0.1% Tween-20 and blocked with 3% BSA in PBS for 1 h at 37°C; 100 µl of immunopurified antigen at different dilutions in PBS were added to the wells and incubated for 1 h at 37°C. Control wells without antigen were incubated with the same buffer. After three washes, 100 µl of $^{125}$I-labelled 83D4 antibody diluted in PBS, 0.1% Tween, 1% BSA (150,000 c.p.m./well) were added to the wells and incubated for 2 h at 37°C. Finally after five washes, wells were cut and bound radioactivity was determined. The $^{125}$I-labelled mAb B72-3 and CC49 were also used as second antibodies under the same conditions as 83D4. Working dilutions of purified antigen were determined by DD-RIA with mAb 83D4 for each sample. All essays were done in duplicate.

*Inhibitory double determinant radioimmunoassay*

Plates were coated with mAb 83D4 or with mAb CC49 as described above for DD-RIA. After blocking with 3% BSA in PBS, antigen was incubated for 1 h at 37°C. After plate washing, 50 µl of $^{125}$I-labelled antibodies (250 ng $ml^{-1}$, corresponding to 150,000 c.p.m. per well) were mixed with increasing concentrations of unlabelled antibodies (ranging from 1 ng $ml^{-1}$ to $10^5$ ng $ml^{-1}$ final concentrations) and added in duplicate to wells. Following 2 h incubation at 37°C, plates were extensively washed with 0.1% Tween 20 in PBS and binding of $^{125}$I-labelled antibody was measured. Percent inhibition as compared to a control buffer sample was determined.

### Results

*Identification of different sources of 83D4 reactive antigen*

MAb 83D4 was generated using paraffin-embedded sections of an invasive breast carcinoma. Flow cytometric analysis of binding of 83D4 to the surface of human breast carcinoma cell lines MCF7, H466B and T47D demonstrated that it was reactive with the three cell lines, the strongest reactivity being with MCF7 cells (Charpin *et al.*, manuscript in preparation). Consequently MCF7 cells were chosen as a source for characterising the 83D4 reactive antigen in breast cancer cells *in vitro*. ELISA performed on cytolsol and membrane fractions of MCF7 cells showed that the antigen was present in the membrane extracts, as expected from previous indirect immunofluorescence studies which showed membrane staining (Pancino *et al.*, 1990*a*). To characterise the antigen expressed in human tissues, 83D4 reactivity with frozen breast cancer tissues extracts was tested using ELISA, and a tumour lysate was chosen among those that showed strong binding with 83D4. Considering the strong staining of colon adenocarcinomas by 83D4 in immunoperoxidase studies of human tissues (Charpin *et al.*, manuscript in preparation), a colon carcinoma extract binding 83D4 in ELISA was chosen as source of 83D4 reactive antigen in a human tumour of origin other than breast. Finally, pleural effusion fluid from patients with metastatic breast carcinomas was tested for reactivity with 83D4 using ELISA, and a reactive sample was chosen to characterise the soluble antigen.

Binding of 83D4 antibody to HMFGM extracts and to skimmed milk was investigated using ELISA. No reactivity was observed with either component of human milk, while strong binding was observed with mAb CA4 and with mAb HMFG1 directed against a human milk fat globule mucin (Figure 1).

*MW determination of antigen*

Analysis of NP40 extracts of MCF7 membranes, breast and colon carcinomas and of pleural effusion fluid was performed by immunoblotting after SDS-PAGE separation on 3–10% polyacrylamide gels. Different methods of sample preparation for SDS-PAGE were used. The best results were obtained using the perchloric acid soluble fraction containing 83D4 reactive antigen. Proteins were precipitated with acetone and solubilised in 9.5 M urea, 4% NP-40, 5% β-mercaptoethanol and 2% SDS. 83D4 reactive antigen was identified as a smear with a range of apparent MW from the origin of gel to about 300 kDa (Figure 2a,d), according to previous results obtained for MCF7 membrane extracts (Pancino *et al.*, 1990*a*). Immunoblotting performed on HMFGM extracts or skimmed milk failed to detect any reactivity with 83D4 (Figure 2c).

The 83D4 reactive antigen was analysed by 2-D gel electrophoresis. Two wide spots were detected by immunoblotting in the acidic area of the gel (data not shown).

*Purification of the 83D4 reactive antigen*

Since pleural effusion fluid was the most abundant source of 83D4-reactive antigen of breast cancer origin available, purification of the antigen was performed first from this material. Immunoreactive material during the different steps of purification was detected by ELISA. The pleural fluid was subjected to perchloric acid (PCA) fractionation and 83D4 reactive material remained in the PCA soluble fraction. PCA soluble material represented about 5% of total proteins. The acid soluble proteins were then subjected to immunoeffinity chromatography with immobilised mAb $83D_4$. For each experiment 10 mg of PCA sample was loaded on the column. Proteins content of the eluate was not quantifiable by Lowry method, but >90% of $83D_4$ antigenic reactivity was recovered in eluted fraction, as assessed by ELISA. Eluted antigen was further purified by size exclusion FPLC in 8 M

**Figure 1** Binding of mAbs 83D4 (10 µg $ml^{-1}$) (▲), HMFG1 (2 µg $ml^{-}$ (□) and CA4 (10 µg $ml^{-1}$) (■) at increasing concentrations of HFMGM extracts, using ELISA. Antibodies were diluted in PBS containing 0.5% gelatine and 0.1% Tween.

**Figure 2** Western blot analysis (3–10% polyacrylamide gel) of 83D4 antigen **a**, **b**, pleural effusion fluid from a breast cancer patient. In **a**, mAb 83D4, in **b**, mAb B1N; **c**, skimmed milk with mAb 83D4, **d**, **e**, breast carcinoma extract: in **d**, mAb 83D4, in **e**, mAb B1N, negative control.

urea buffer to ensure complete solubilisation of the proteins. The immunoreactive material eluted in the void volume of the column, indicating the high MW of the antigen (Figure 3).

Lower molecular weight proteins, coeluted with the antigen from the immunoaffinity column, did not bind 83D4. The reactive fractions were pooled and analysed by anion exchange FPLC. ELISA performed on the eluted fractions showed multiple reactive peaks, evidencing the charge heterogeneity of the antigen (Figure 4). The antigenic activity profile was similar to the 280 nm absorbance pattern, thus

**Figure 3** Size exclusion chromatography of 83D4 immunoaffinity purified antigen from a pleural effusion fluid on FPLC with Superose-6 HR column. Flow rate: 0.5 ml min$^{-1}$. The eluate was monitored in absorbance at 280 nm and the collected fractions were tested for 83D4 reactivity in ELISA.

**Figure 4** Anion exchange on FPLC with a mono-Q column of the pooled 83D4 reactive peak from size exclusion column. Gradient: 0–0.5 M NaCl. Flow rate of 0.8 ml min$^{-1}$. Eluted fractions were assayed for 83D4 reactivity in ELISA.

indicating that a good antigen purification degree has been obtained. 83D4 antigen was also isolated from $MCF_7$ membrane extracts, using the same method.

### *Lectin binding assays*

The immuno/FPLC (Vo) purified 83D4 reactive antigen from the pleural effusion fluid was incubated with 4 Sepharose-CL4B immobilised lectins with different carbohydrate specificities: WGA, CONA, Peanut and Lentil. After absorption, supernatants were tested by ELISA for residual antigenic reactivity with 83D4 (not shown). No significant reduction in 83D4 binding compared with the Sepharose CL4B sample was found with CONA and Lentil, a weak reduction was observed with PNA while WGA incubation abolished sample reactivity. After elution with appropriate carbohydrates no antigenic material was found using ELISA in eluates from CONA and Lentil and weak $83D_4$ reactivity was observed with Peanut eluate. Conversely $83D_4$ reactivity was completely recovered in eluate from WGA (Figure 5).

### *Biochemical analysis of 83D4 reactive antigen*

The immunopurified antigen was subjected to proteolytic digestion with trypsin and subtilysin, resulting in a sharp reduction in 83D4 binding. Figure 6a shows that trypsin gradually reduced 83D4 reactivity up to 90% at a 2.5 mg ml$^{-1}$ concentration, while subtilysin had already abolished antibody binding at the first concentration tested (0.125 mg ml$^{-1}$).

To determine whether carbohydrate structures take part in the 83D4 antigenic determination, the antigen was subjected to sodium periodate oxidation. Figure 6b shows that 83D4 binding was reduced after Na periodate treatment, up to an 85% reduction at 20 mM metaperiodate concentrations. In contrast, antigenic reactivity was not affected by treatment with Neuraminidase from *Vibrio cholera* or from *Clostridium perfringens* (Figure 6c).

### *Repeated epitope expression of 83D4 reactive antigen*

Immunoaffinity-purified antigen was tested for its reactivity in a double determinant radioimmunoassay. Antigen purified from breast cancer pleural effusion and colon carcinoma

**Figure 5** Lectin binding to 83D4 immuno/FPLC (Vo) purified antigen from a pleural effusion fluid. After incubation with different lectins (WGA (◆), CONA (▲), Lentil (◇), Peanut (■)), bound antigen was eluted with appropriate sugars as described in 'Materials and methods'. Recovered antigenic activity was assessed by ELISA with mAb 83D4.

tissue was incubated in microplates coated with mAb 83D4; $^{125}$I-labelled 83D4 antibody binding was subsequently measured on fixed antigen. Significant binding of the radiolabelled antibody (Figure 7a,b) indicated that the antigen expressed multiple 83D4 reactive epitopes.

### *HMFG1 reactivity with the 83D4 defined antigen*

To investigate the expression of HMFG1 epitope on $83D_4$ defined antigen an ELISA was performed using the immuno/FPLC (Vo) purified $83D_4$ antigen from $MCF_7$ membrane extracts. No significant reactivity was detected with HMFG1, which conversely bound to the $MCF_7$ PCA sample from which antigen had been isolated (Figure 8). Immunoblotting of $MCF_7$ PCA sample and purified antigen confirmed these results (not shown).

### *B72-3 and CC49 reactivity with 83D4 defined antigen*

MAb B72-3 directed against a high MW carcinoma-associated mucin TAG-72, and the second-generation mAb CC49 raised to purified TAG-72 were tested for reactivity to the $83D_4$ defined antigen. ELISA assays on the immuno/FPLC (Vo) purified $83D_4$ antigen from $MCF_7$ membranes or pleural effusion fluid showed CC49 binding to the antigen, while very weak reactivity was detected with B72-3 (not shown). Moreover FPLC filtration fractions of immunopurified $83D_4$ antigen were tested for reactivity with mAbs $83D_4$ and CC49: antigenic activity was found in the Vo fraction as expected, while low molecular species were unreactive with both antibodies. Immunoblotting of PCA sample from a pleural effusion fluid was then performed to compare reactivity patterns of mAbs $83D_4$ and CC49. Reactive bands of high MW with similar electrophoretic mobility were detected by the two antibodies (Figure 9). In further RIA experiments immunopurified antigens were used. $83D_4$ antigens from pleural effusion fluid, breast carcinoma and colon carcinoma extracts were tested in a double determinant RIA for binding to mAb B72-3 and CC49. Table I shows that $^{125}$I-labelled B72-3 and CC49 displayed binding with 83D4 antigen fixed on mAb 83D4-coated plates with differential reactivity depending on the source of the antigen. B72-3 showed weak binding to the 83D4 defined antigen immunopurified from pleural effusion, colon or breast carcinoma. CC49 exhibited weak reactivity with breast carcinoma or pleural effusion antigen, but a strong reaction with the colon carcinoma antigen.

83D4, B72-3 and CC49 were analysed in a inhibitory double determinant RIA on pleural effusion and colon carcinoma immunopurified antigens in order to study the rela-

**Figure 6a** Effect of proteolytic digestion of 83D4 immunopurified antigen from a pleural effusion fluid on 83D4 reactivity. Antigen coated on microplates was incubated with increasing concentrations of trypsin (□) or subtilysin (■) as described in 'Materials and methods'. After treatment, 83D4 binding was determined by ELISA. **b**, Effect of Na-metaperiodate treatment on 83D4 reactivity with the immunopurified antigen from a pleural effusion. Antigen absorbed on a microplate was exposed to increasing concentrations of periodate as described in 'Materials and methods'. After treatment, 83D4 reactivity was tested by ELISA. **c**, Neuraminidase digestion of 83D4 defined antigen immunopurified from a pleural effusion. Antigen absorbed on a microplate was treated with increasing concentrations of neuraminidase from *Vibrio cholera* (□) or from *Clostridium perfringens* (■). After digestion, 83D4 reactivity was tested by ELISA.

**Figure 7** Repeated epitope expression of 83D4 defined antigen. Double determinant-RIA was performed as described in 'Materials and methods' on serial dilutions of antigen immunopurified from **a**, a breast cancer pleural effusion fluid or **b**, a colon carcinoma extract.

**Figure 8** HMFG1 reactivity with 83D4 antigen; **a**, ELISA on immuno/FPLC (Vo) purified 83D4 antigen from MCF7 membranes extract; **b**, ELISA on PCA fraction of MCF7 membranes extract from which 83D4 antigen used in *a*, was isolated. (HMFG1 (□), 83D4 (▲), and B1N (△). MAbs concentration: 10 $\mu g\ ml^{-1}$).

tionship between antigenic determinants of these antibodies. Similar results were found with antigens of both origins. No competition with 83D4 was shown by mAb B72-3 (results not shown). CC49 competed at 35% for the highest concentrations (50 and 100 $\mu g\ ml^{-1}$ of CC49) for the colon-cancer-derived antigen (Figure 10a). In the reciprocal mAb CC49 competition RIA, 83D4 competed at 65% for maximal concentrations (Figure 10b).

### Discussion

MAb 83D4 was produced by immunising mice with paraffin sections of an invasive breast carcinoma. It has previously been shown to react with both fixed and paraffin-embedded and frozen breast carcinoma tissues (Pancino *et al.*, 1990*a*). No reactivity was detected with normal breast. These studies indicated that 83D4 identifies a native antigen not affected by fixation and paraffin embedding procedures, and that the antigen is well expressed in breast cancer, but is absent in normal mammary epithelium.

In the present work, the antigen was detected in MCF7 breast cancer cell line membrane extracts, in primary breast carcinoma and in colon carcinoma extracts. The antigen was also detected and then isolated from a pleural effusion fluid of patients with metastatic breast carcinoma, indicating that it can be released in soluble form from breast cancer cells. No detectable expression of the antigen was found in human milk components. Immunoblotting analysis showed that 83D4 epitopes are carried by heterogeneous acidic proteins of high MW (>300 kDa). The antigen, soluble in PCA, was purified using immunoaffinity chromatography, subsequent gel filtration and anion exchange chromatography. The 83D4-reactive molecules, solubilised in 8M urea buffer, were eluted in the void volume of a Superose 6 FPLC gel column and resolved in multiple peaks by anion exchange FPLC. The immunopurified 83D4-reactive antigen bound to WGA, specific for N-acetyl – D-glucosamine and sialic acid, and weaker to Peanut lectin. No binding was observed with other lectins (CONA, Lentil). The antigenic activity was affected by protease digestion with trypsin and subtilysin. These findings taken together indicate that the antigen is composed of heterogeneous acidic glycoproteins of high MW and that the carbohydrate component contains N-acetyl glucosamine and/or sialic acid. The biochemical nature of the 83D4 reactive epitope was further analysed by neuraminidase digestion and sodium periodate oxidation. The gradual decrease in 83D4 binding upon periodate oxidation from 0.1 mM to 20 mM periodate concentrations suggests that the epitope defined by 83D4 involves carbohydrates.

However the antigen was resistant to digestion with neuraminidase from *Vibrio cholera* and *Clostridium perfringens*, suggesting that sialic acid is not required for binding of 83D4.

We looked for a relationship of 83D4 defined antigen with other breast cancer associated high MW glycoproteins defined by murine mAbs. A well known 'immunodominant' antigenic family is a mucin found in human milk, breast cancer and in other tumoral and normal epithelia, and identified by several mAb (Taylor-Papadimitriou *et al.*, 1981;

**Figure 9** Immunoblotting of PCA sample from a pleural effusion fluid, with mAbs 83D4 (lane **a**) and CC49 (lane **b**). Lane c: mAb 1BE12, negative control.

**Figure 10** Inhibitory double determinant-RIA using $^{125}$I-labelled mAb 83D4 **a**, and $^{125}$I-labelled mAb CC49, **b**. Purified mAb 83D4 and CC49 were used as competitors for binding of the radiolabelled mAb with the 83D4 defined antigen purified from a colon carcinoma extract as described in 'Materials and methods'. Concentrations of competitor antibody are expressed in ng ml$^{-1}$ **a**, 83D4 (□), CC49 (■), **b**, CC49 (□), 83D4 (■).

**Table I** Reactivity of mAb 83D$_4$ and mAbs B72-3 and CC49 with 83D4 defined antigen purified from three different sources using S-RIA

| *Antigen source* | *83D4** | *B72-3* | *CC49* |
|---|---|---|---|
| Pleural effusion | 6172** | 1115 | 2819 |
| Breast carcinoma | 13315 | 588 | 2340 |
| Colon carcinoma | 16214 | 1259 | 20950 |

Buffer controls: 83D4 504 c.p.m., B72-3 199 c.p.m., CC49 229 c.p.m.; *specific activity of $^{125}$I-labelled antibodies: 83D4: 3.70 μCi μg$^{-1}$; B72-3 and CC49: 3.25 μCi μg$^{-1}$; **c.p.m. bound.

Ashall *et al.*, 1982; Hilkens *et al.*, 1984; Papsidero *et al.*, 1984; Sekine *et al.*, 1985; Price *et al.*, 1986; Linsley *et al.*, 1986; Stacker *et al.*, 1989). cDNA clones coding for the core protein of this mucin, termed Polymorphic Epithelial Mucin (PEM), were recently isolated and the amino acid sequence was fully determined (Gendler *et al.*, 1988 and 1990). We used mAb HMFG1, which defines the PEM antigen, to study the relationship with 83D4 antigen: no binding of HMFG1 to 83D4 purified antigen could be detected by ELISA and immunoblotting.

The glycoprotein nature of the antigen recognised by 83D4, its binding to WGA, its apparent MW and heterogeneity and its selective reactivity for breast tumours *vs* normal breast tissue are similar to the characteristics of a pancarcinoma-associated glycoprotein TAG-72 (Johnson *et al.*, 1986) defined by mAb B72.3, generated using a membrane preparation from a mammary carcinoma metastasis (Colcher *et al.*, 1981). It was thus important to determine whether a relationship of 83D4 antigen to TAG-72 exists. MAbs B72.3 and colon cancer-purified TAG-72 second generation mAb CC49 (Muraro *et al.*, 1988) were used for this study. 83D4 immuno/FPLC (Vo) purified antigen bound the two antibodies in ELISA and immunoblotting showed similar reactivity patterns of 83D4 and CC49 antibodies. Since the reactivity of mAb B72.3 and CC49 has been shown to vary with different tumour extracts of breast and colon carcinomas (Muraro *et al.*, 1988), three different sources of 83D4 defined antigen were used for further cross-reactivity studies. Differential binding of mAb B72.3 and CC49 to 83D4 defined antigen was found using the antigen purified from pleural effusion fluid, breast carcinoma or colon carcinoma. B72.3 bound weakly to effusion fluid and colon-carcinoma-derived antigens and very weakly to the breast-carcinoma-derived antigen, while CC49 bound weakly to antigens of breast cancer origin, but stronger to the colon-cancer-derived antigen. These results indicate that 83D4 defined antigen carries epitopes reactive with B72.3 and CC49, which may vary quantitatively in different sources, CC49 epitope being most expressed in colon carcinoma. MAb B72.3 and CC49 both react with the carbohydrate epitopes (Johnson *et al.*, 1986; Kjeldsen *et al.*, 1988; Sheer *et al.*, 1988) and the 83D4 epitope also appears to involve sugar moieties. A competition RIA showed that B72.3 did not compete with 83D4, and that CC49 only partially inhibited 83D4 binding to its antigen. The failure of B72.3 to compete with 83D4 could be expected, since the B72.3-reactive epitope has been shown to be a sialosyl-Tn structure (Kjeldsen *et al.*, 1988) sensitive to neuraminidase digestion (Kjelden *et al.*, 1988 & Johnson *et al.*, 1986), while the 83D4 epitope was not affected by neuroaminidase treatment. There are several possible interpretations for the partial competition between CC49 and 83D4: the two epitopes may be distinct, but in physical proximity to each other; they may be only partially overlapping; or partial inhibition may have resulted from conformational changes induced by antibody binding. It is possible that conformational changes in the antigen molecule or steric hindrance of the binding site caused by 83D4 IgM immunoglobulin may partially account for the asymmetric pattern of cross-competition between IgM 83D4 and IgG1 CC49 (CC49, at the highest concentrations, inhibited 35% of 83D4 binding to antigen; 83D4 inhibited 65% of CC49 binding in reciprocal competition RIA).

In the summary, the results reported above suggest that the antigen identified by 83D4 shares common antigenic activity with the TAG-72 antigen identified by B72.3 and CC49, but expression of reactive epitopes varies with the antigen source. The antigenic determinant identified by 83D4 appears to be different from those defined by B72.3 and CC49. The relationship shown to exist between the 83D4 reactive antigen and the TAG-72 antigen is suggestive of the hypothesis that they may belong to the same family of related, high MW tumour-associated glycoproteins. The difference in immunoreactivity found with 83D4 in comparison with B72.3 or CC49 may result from expression of a different number of carbohydrate epitopes on the same protein molecule, caused by varying degrees of glycosylation. However, it cannot be excluded that differential reactivity may be due to different proteins carrying the epitopes on the oligosaccharide side chains. It is worth noting that hybridoma-secreting mAb 83D4 was generated in a completely different way from B72.3 and CC49. Epitope analysis and mapping of 83D4 defined antigen and TAG-72 antigen, as well as cloning of coding genes, would clearly define their relationship and indicate whether these molecules constitute a new family of carcinoma-associated glycoprotein antigens. At present, it is known that mAb B72.3 identifies a sialyl-Tn epitope, and CC49 has been claimed to react with other oligosaccharides; this study demonstrates that 83D4 identifies a novel epitope and that sugar moieties take part in it. A number of tumour-associated glycoprotein carbohydrate epitopes have been detected by mAb, and some of them have been characterised, including antigen reactive with the mAb 19-9 directed to a sialyl-Le$^a$ carbohydrate chain carried on a gastrointestinal-cancer-associated mucin (Magnani *et al.*, 1983), and the Tn antigen identified by mAb NCC-LU-35 and NCC-LU-81 raised against squamous lung carcinomas (Hirohashi *et al.*, 1985). Aberrant glycosylation of glycoprotein antigens is a common phenomenon in human cancers (reviewed in Hakomori, 1989). Studies to define the structure of the 83D4 reactive epitope and its function are now required to elucidate its relationship with oncogenic transformation.

We thank Dr J. Schlom (N.C.I., Meryland) for the gift of mAbs B-72.3 and CC49, Dr J. Taylor-Papadimitriou for the gift of mAb HMFG1, M. Beuzelin for providing pleural effusion fluids, P. Mouly (C.I.S. Bioindustries) for help in labelling mAbs with $^{125}$I, J. Boussange (Pharmacia France) for technical assistance in FPLC experiments, Dr M.H. Toubert and M.H. Schlageter for helpful discussion, B. Boursin for photographical work and C. Guilbert for secretarial assistance. This work was supported in part by the Ministère de la Recherche et de la Technologie and the Association pour la Recherche sur le Cancer.

### References

ASHALL, F., BRAMWELL, M.E. & HARRIS, H. (1982). A new marker for human cancer cells. I. The CA antigen and the Ca1 antibody. *Lancet*, **ii**, 1.

BURCHELL, J., GENDLER, S., TAYLOR-PAPADIMITRIOU, J. & 4 others (1987). Development and characterization of breast cancer reactive monoclonal antibodies directed to the core protein of the human milk mucin. *Cancer Res.*, **47**, 5476.

COLCHER, D., HORAN HAND, P., NUTI, M. & SCHLOM, J. (1981). A spectrum of monoclonal antibodies reactive with human mammary tumor cells. *Proc. Natl Acad. Sci USA*, **78**, 3199.

DELONG, S. & DAVIDSON, E. (1981). Preparation and properties of a glycoprotein associated with malignancy. *Biochemistry*, **20**, 1047.

FRANKEL, A.E., RING, D.B., TRINGALE, F. & HSIEH-MA, S.T. (1985). Tissue distribution of breast cancer-associated antigens defined by monoclonal antibodies. *J. Biol. Resp. Modif.*, **4**, 273.

GARCIA-GONZALES, M., BETTINGER, S., OTHI, S., OLIVER, P., KADOUCHE, J. & POULETTY, Ph. (1988). Purification of murine $IgG_3$ and IgM monoclonal antibodies by englobulin precipitation. *J. Immunol. Methods*, **111**, 18.

GENDLER, S., TAYLOR-PAPADIMITRIOU, J., DUHIG, T., ROTHBARD, J. & BURCHELL, J. (1988). A highly immunogenic region of a human polymorphic epithelial mucin expressed by carcinomas is made of tandem repeats. *J. Biol. Chem.*, **263**, 12820.

GENDLER, S.J., LANCASTER, C.A., TAYLOR-PAPADIMITRIOU, J. & 6 others (1990). Molecular cloning and expression of the human tumor-associated polymorphic epithelial mucin, PEM. *J. Biol. Chem.*, **266**, 12286.

HAKOMORI, S. (1989). Aberrant glycosylation in tumors and tumor-associated carbohydrate antigen. *Adv. Cancer Res.*, **52**, 257.

HILKENS, J., BUIJS, F., HILGERS, J. & 4 others (1984). Monoclonal antibodies against human milk fat globule membranes detecting differentiation antigens of the mammary gland and its tumors. *Int. J. Cancer*, **34**, 197.

HIROHASHI, S., CLAUSEN, H., YAMADA, T., SHIMOSATO, Y. & HAKOMORI, S. (1985). Blood group A cross-reacting epitope defined by monoclonal antibodies NCC-LU-35 and -81 expressed in cancer of blood group O or B individuals: its identification as Tn antigen. *Proc. Natl Acad. Sci USA*, **82**, 7039.

JOHNSON, V.G., SCHLOM, J., PATERSON, A.J., BENNET, J., MAGNANI, J.L. & COLCHER, D. (1986). Analysis of a human tumor-associated glycoproteins (TAG-72) identified by monoclonal antibody B72.3. *Cancer Res.*, **46**, 850.

KEENAN, T.W., MORRE, J.D., OLSON, D.E., KUNGHANS, W.N. & PATTON, S. (1970). Biochemical and morphological comparison of plasma membrane and milk fat globule membrane from bovine mammary gland. *J. Cell. Biol.*, **44**, 80.

KEYDAR, I., CHOI, C.S., HAREUVENI, M. & 5 others (1989). Production and characterization of monoclonal antibodies identifying breast tumor-associated antigens. *Proc. Natl Acad. Sci. USA*, **86**, 1362.

KJELDSEN, T., CLAUSEN, H., HIROHASHI, S., OGAWA, T., IIJIMA, H. & HAKOMORI, S. (1988). Preparation and characterization of monoclonal antibodies directed to the tumor-associated o-linked sialyosyl 2–6 Nacetylgalactosaminyl (sialosyl-Tn) epitope. *Cancer Res.*, **48**, 2214.

LAEMLI, U.K. (1970). Cleavage of structural proteins during the assembly of the head of bacteriophage T4. *Nature*, **227**, 680.

LINSLEY, P.S., OCHS, V., LASKA, S. & 4 others (1986). Elevated level of a high molecular weight antigen detected by antibody W1 in sera from breast cancer patients. *Cancer Res.*, **46**, 5444.

LOWRY, O., ROSEBROUGH, N., FARR, A. & RANDALL, R. (1951). Protein measurement with the Folin phenol reagent. *J. Biol. Chem.*, **193**, 265.

MAGNANI, J.L., STEPLEWSKI, Z., KOPROWSKI, H. & GINSBURG, V. (1983). Identification of the gastrointestinal and pancreatic cancer-associated antigen detected by monoclonal antibody 19-9 in the sera of patients as a mucin. *Cancer Res.*, **43**, 5489.

MURARO, R., KUROKI, M., WUNDERLICH, D. & 8 others (1988). Generation and characterization of B72.3 second generation monoclonal antibodies reactive with the tumor-associated glycoprotein 72 antigen. *Cancer Res.*, **48**, 4588.

PANCINO, G., CHARPIN, C., CALVO, F., GUILLEMIN, M.-C. & ROSETO, A. (1987). A novel monoclonal antibody (7B10) with differential reactivity between human mammary carcinoma and normal breast. *Cancer Res.*, **47**, 4444.

PANCINO, G., LE DOUSSAL, V., MORTADA, M.H. & 4 others (1989). Characterization and distribution in normal and tumoral human tissues of breast cancer-associated antigen defined by monoclonal antibody 7B10. *Cancer Res.*, **49**, 7078.

PANCINO, G., OSINAGA, E., VORAUHER, W. & 4 others (1990*a*). Production of a monoclonal antibody as an immunohistochemical marker on paraffin embedded tissues using a new immunization method. *Hybridoma*, **9**, 389.

PANCINO, G., CHARPIN, C., OSINAGA, E. & 4 others (1990*b*). Characterization and distribution in human tissues of a glycoproteic antigen defined by monoclonal antibody $1BE_{12}$ raised against T47D human breast cancer cell line. *Cancer Res.*, **50**, 7333.

PANCINO, G., MORTADA, M.H., CHARPIN, C. & 6 others (1991). Two monoclonal antibodies identify antigens preferentially expressed on normal human breast cells versus breast cancer cells. *Hybridoma*. (in press).

PAPSIDERO, I.D., CROGHAN, G.A., JOHNSON, E.A. & CHU, T.H. (1984). Immunoaffinity isolation of ductal carcinoma antigen using monoclonal antibody F 36/22. *Mol. Immunol.*, **21**, 955.

PRICE, M.R., EDWARDS, S., POWELL, M. & BALDWIN, R.W. (1986). Epitope analysis of monoclonal antibody NCRC-11 defined antigen isolated from human ovarian and breast carcinomas. *Br. J. Cancer*, **54**, 593.

SEKINE, H., OHNO, T. & KUFE, D.W. (1985). Purification and characterization of high molecular weight glycoprotein detectable in human milk and breast carcinomas. *J. Immunol.*, **135**, 3610.

SHEER, D.G., SCHLOM, J. & COOPER, H.L. (1988). Purifiction and composition of the human tumor-associated glycoprotein (TAG-72) defined by monoclonal antibodies CC49 and B72.3. *Cancer Res.*, **48**, 6811

SOULE, H.D., VASQUEZ, J., LANG, A., ALBE, L.S. & BREMAN, M. (1973). A human cell line from a pleural effusion derived from a breast cancer. *J. Natl Cancer*, **31**, 1409.

STACKER, S.A., FJANDRA, J.J., PEI-XIANG XING, I.D., WALKER, I.D., THOMPSON, C.H. & MCKENZIE, I.F.C. (1989). Purification and biochemical characterization of a novel breast carcinoma associated mucin-live glycoprotein defined by antibody 3E1.2. *Br. J. Cancer*, **59**, 544.

TAYLOR-PAPADIMITRIOU, J., PETERSON, J., ARKLIE, J., BURCHEL, J., CERIANI, R.L. & BALMER, W. (1981). Monoclonal antibodies to epithelium specific components of the human milk fat globule membrane: production and reaction with cells in culture. *Int. J. Cancer*, **28**, 17.

THOR, A., OHUCHI, N., SZPAK, C.A., JOHNSTON, W.W. & SCHLOM, J. (1986). Distribution of oncofetal antigen tumor-associated glycoprotein – 72 defined by monoclonal antibody B72-3. *Cancer Res.*, **46**, 3118.

TOWBIN, H., STAEHLIN, T. & GORDON, J. (1979). Electrophoretic transfer of proteins from polyacrylamide gels to nitrocellulose sheets: procedure and some applications. *Proc. Natl Acad. Sci. USA*, **76**, 4350.

WOODWARD, M.P., YOUNG, W.W. & BLOODGOOD, R.A. (1985). Detection of monoclonal antibodies specific for carbohydrate epitope using periodate oxidation. *J. Immunol. Methods*, **78**, 143.

WRESCHNER, D.H., HAREUVENI, M., TSARFATY, I. & 8 others (1990). Human epithelial tumor antigen cDNA sequences. *Eur. J. Biochem.*, **189**, 463–473.